# The relationship between reproductive outcome measures in DDT exposed malaria vector control workers: a cross-sectional study

**DOI:** 10.1186/1745-6673-1-21

**Published:** 2006-08-10

**Authors:** Mohamed A Dalvie, Jonathan E Myers

**Affiliations:** 1 Occupational and Environmental Health Research Unit, School of Public Health and Family Medicine, Faculty of Health Sciences, University of Cape Town, Anzio Road, Observatory, 7925, Cape Town, South Africa

## Abstract

**Background:**

The utility of blood reproductive endocrine biomarkers for assessing or estimating semen quality was explored.

**Methods:**

A cross-sectional study of 47 DDT exposed malaria vector control workers was performed. Tests included blood basal and post gonadotrophin releasing hormone (GnRH), lutenizing hormone (LH), follicle stimulating hormone (FSH), testosterone, sex hormone binding globulin (SHBG), estradiol (E2) and inhibin; a questionnaire (demographics and general medical history); a physical examination and semen analysis. Semen parameters were determined using either/or or both WHO or the strict Tygerberg criteria. Relationships between semen parameters and endocrine measures were adjusted for age, duration of abstinence before sampling, presence of physical abnormalities and fever in the last two months. All relationships between specific endocrine hormones were adjusted for age and basal SHBG.

**Results:**

Multiple logistic regression showed a consistent positive relationship (prevalence odds ratio (POR) = 8.2, CI:1.4–49.2) between low basal inhibin (<100 pg/ml) and low semen count (< 40 million) and density (< 20 million/ml); consistent positive, but weaker relationships (1> POR < 2) between abnormally low semen count as well as density and baseline and post GnRH FSH; and positive relationships (POR = 37, CI:2–655) between the prevalence of high basal estradiol (> 50 pg/ml) and abnormal morphology (proportion < 5%) and low motility (proportion <50%). Most of the expected physiological relationships between specific endocrines were significant.

**Conclusion:**

The study has demonstrated that low basal inhibin, elevated basal FSH and high basal E2 can serve as markers of impaired semen quality.

## Background

Semen quality parameters are routine clinical measures used to assess testicular function and basal blood reproductive endocrine levels are used to assess the integrity of the hypothalamus-pituitary-testicular axis. The GnRH stimulation test has also been used to indicate disruption in the normal hypothalamic-pituitary-testicular axis (HPT) [1,2] in men with significant gonadal dysfunction due to testicular disorders such as cryptorchidism, varicocele, testicular torsion and vasectomy [2].

A number of epidemiological studies have investigated the relationships between semen quality and endocrine measures mostly in men diagnosed with infertility [1,3-12]. Basal inhibin has been shown to be predictive of semen quality having positive relationships with most semen parameters, especially semen count [3-5,7,9]. There is also evidence that basal FSH has a negative relationship with semen quality [3,8,10]. Gerhard et al. [12] did not find significant relationships between post GnRH challenge hormone levels and semen parameters. Few epidemiological studies have investigated relationships between individual blood reproductive endocrine levels [1,3,5,11]. A negative relationship between basal inhibin and basal FSH was the only physiological relationship consistently found. Besser [1] did not find relationships between basal and post GnRH challenge blood hormone levels.

No studies could be found in the literature that have comprehensively examined to what extent blood endocrines predict semen parameters or examined relationships between individual blood endocrines in humans. Although most previous studies controlled for factors such as abstinence and age through the selection of study subjects for relationships between semen parameters and blood endocrines, a limitation was that other covariates was not controlled for. This study examines the relationship between a number of semen parameters and endocrine measures as well as relationships between individual blood endocrines controlling for relevant covariates, in malaria control workers investigated for the reproductive effects of DDT where no DDT effect was found [13,14].

## Methods

### Subjects, questonnaire and physical examination

The details of the study methods are described elsewhere [3-15]. Briefly, a cross-sectional study of Pedi-speaking workers (n = 47) from three camps in the vicinity of the Department of Health Malaria Control Centre (MCC) in Tzaneen, Limpopo, was performed.

A questionnaire [13] including amongst others, sections on demographics and general medical history was administered by trained interviewers. A doctor performed an abbreviated physical examination of the reproductive system recording height, weight, secondary sexual characteristics (Tanner stage) and genital anatomical abnormalities. The presence of infection, previous injury, hernias or tumours were assessed.

### Endocrine measures

Baseline and post-GnRH (100 μg) challenge test levels of pituitary and gonadal hormones FSH, LH, testosterone, estradiol (E2), SHBG and Inhibin-B were measured. The Department of Chemical Pathology at the University of Cape Town measured LH with the MAIAclone IRMA kit [16,17], FSH and total testosterone with ACS-180 competitive chemiluminescent automated systems [18], estradiol with an in-house radioimmunoassay [19] and SHBG with the IRMA kit from Orion Diagnostica [20]. Laboratory inter- and intra-assay variation was less than 6.8% and 13% respectively [15]. Inhibin B was measured by the Centre for Reproductive Biology, Medical Research Council, Edinburgh, United Kingdom using an internally validated two site enzyme-linked immunoassay [21-23]. Baseline hormones were compared to manufacturers reference values, while for SHBG, the laboratory's internal reference range (12.7–55 nmol/L) was used because it differed from the manufacturers' range (11–71 nmol/L).

### Semen quality

Workers were requested to produce semen samples by masturbation or coitus interruptus (which was more culturally compatible with the beliefs and practices of participants) after 2 days of abstinence, one hour before collection time, and to keep them at body temperature. Collected samples were then immediately transported (at room temperature) to the MCC laboratory and incubated at room temperature. An experienced reproductive biologist performed analysis including semen volume (to the nearest 0.1 ml in a graded tube); sperm count (millions) diluted 1:20 with formalin buffer in an improved Neubauer hemacytometer and using the phase-contrast technique at a magnification of 40; sperm density (millions/ml); quantitative sperm motility (% motile relative to immobile sperm, estimated to the nearest 5%) at a magnification of 20 using an undiluted semen sample of ≤ 10 μl ; as well as liquefaction, consistency, pH, and agglutination, following World Health Organisation protocols [24]. Air-dried slides were air shipped to the Department of Obstetrics and Gynaecology at the University of Cape Town for morphology determination (% normal) using the strict Tygerberg criteria [25]. Slides were fixed in 80% alcohol and stained using a modification of Papanicolou's stain. A phase contrast light microscope was used for semen analysis.

### Statistical analysis

Semen parameters and endocrine measures were analysed as both continuous variables and dichotomous variables. Dichotomous cut-offs for semen parameters were based on WHO (density < 20 million/ml, count < 40 million and motility < 50%) [24] and/or Tygerberg (morphology < 2%) criteria [25], while basal endocrines were dichotomised at upper and lower limits of the reference ranges and post-GnRH challenge hormones at zero, upper and lower quartiles. Blood hormone responses to GnRH, were analysed as the absolute change at different time points from the mean of the two pre-challenge baseline measures. The peak change across all timepoints and the summed change over all timepoints were calculated. Univariate, bivariate and multivariate analyses, using multiple linear and multiple logistic regression analysis, were performed for relevant variables. The relationship between semen parameters and endocrine measures on the one hand, and between basal and post GnRH endocrine levels on the other were explored. In multivariate modeling, semen parameters were treated as outcomes for relationships with endocrine levels adjusting for age, abstinence from sex, the presence of structural or pathological abnormalities and fever in the last 2 months, while basal hormone levels were outcomes for relationships with post GnRH challenge levels of the same hormone adjusting for age and basal SHBG. Covariates were selected a priori based on biological plausibility, or were based on bivariate analysis yielding associations where the p-value was < 0.1. DDT was not a significant covariate. Analysis was conducted using Stata 8 [26]. Multiple linear regression models were evaluated for normality of residuals, homogeneity of variances, and collinearity. Multiple linear and logistic regression models were tested for the form of the linear predictor and for the adequacy of the link function. Where there was evidence of skewness in the distribution of the residuals for multiple linear regression, this was alleviated by logarithmic transformations, but because the transformed models did not change the nature of associations, the untransformed models are presented. For multiple linear regression analyis, collinearity was identified if r > 0.9 or a variance inflation factor > 10, and the effect of outliers/influential points, identified by DFBETAs > 1, Cook's D > 0.5 or Student residuals > 2.5 while outliers and influential points for multiple logistic regression analysis were identified if standardized residuals were > 2 or < -2 or if leverage patterns were far from the average covariate pattern. Outliers and influential points did not have an effect on the nature of the multivariate associations and the results of relationships including all points are therefore presented. The adjusted R^2 ^indicates the proportion of the total variance explained by a multivariate model after adjusting for the number of variables in the model. The term "R^2^" is, however, used to avoid confusion with term "adjusted effect" and "adjusted ".

### Ethical approval

The study was conducted in accordance with national and institutional guidelines for the protection of human subjetcs. The study protocol was approved by the University of Cape Town's Ethics Committee and by the University of Michigan Internal Review Board. Written informed consent was obtained from workers whose confidentiality was preserved.

## Results

### Descriptive results

Descriptive results are described in detail elsewhere [13,14]. Briefly the participants had a high mean age of 43.3 (SD = 9.0 years). The prevalence of abnormal semen (any abnormality) amongst the participants was high (> 45%). Median baseline LH, FSH, SHBG and testosterone of workers were within normal (laboratory reference) ranges, while median baseline E2 was above the upper limit (50 pg/ml) of normal. Sixty five percent of participants' baseline E2s, 2% LH and FSH, 14% testosterone, 18% SHBGs and no inhibin were above the upper limit of normal, while 23% baseline LH and FSH, 2% E2 and SHBG and 4% testosterone and 45% inhibin were below the lower limit of normal. As indicated in Table 1, post GnRH responses of LH and FSH were well above baseline values, while post GnRH testosterone, inhibin and estradiol responses were much less and sometimes negative.

**Table 1:** Comparison of blood hormone levels of participants with normal and abnormal sperm

Variable	Median (range)							
	
	Semen count		Semen density		Semen Motility		Normal semen morphology	
	
	≥ 20 × 10^6 ^(n = 26)	< 20 × 10^6 ^(n = 17)	≥ 40 × 10^6^/ml (n = 31)	< 40 × 10^6^/ml (n = 14)	≥ 50% (n = 29)	< 50% (n = 14)	≥ 2% (n = 25)	< 2% (n = 16)
Basal:								
LH (mui/ml)	1.8 (0.7;4.7)	2.7 (1.3;11.7)	2.3 (0.7;4.7)	2.2 (1.3;11.7)	2.1 (0.7;4.6)	2.5 (1.4;6.2)	2.3 (0.7;4.6)	2.2 (0.7;6.2)
FSH(mui/ml)	2.7 (0.1;2.2)	3.9 (0.8;19.9)	2.5 (0.1;7.2)	3.9 (1.7 ;19.9)	2.7 (0.1;7.3)	3.6 (0.1;8.5)	2.7 (0.1;2.2)	2.7 (1.1;8.5)
TST (nmol/L)	18.4 (8.0;39.5)	17.0 (9.3;39.4)	20.2 (4.7;39.5)	15.8 (9.3;32.1)	16.4 (4.7;39.4)	20.6(13.7;39.5)	17 (4.7;32.5)	18.1(9.7;32.1)
E2 (pg/ml)	53.5 (2.0;208.5)	55.0 (13.5;115.5)	53.5 (2.0;208.5)	55.0 (15.0;115.5)	53.5 (2.0;122)	73 (36;208.5)	48.3 (2;208.5)	69.3 (50;159.5)
								
IHB (pg/ml)	121.1(33.4;231.3)	73.3(25.2;192.2)	122.0 (35.4; 244.7)	81.4 (25.2;164.1)	115.3 (35.4;244.7)	133.8 (50.1;202.8)	123.3 (35.4;244.7)	101.4 (37.9;173.1)
SHBG (nmol/L)	32.8 (15.5;95.0)	37.0 (12.0;76.5)	33.5 (15.5; 95.0)	35.5 (12.0;76.5)	33.0 (12.0;95.0)	37.8 (23.5;65.5)	34 (15.5;95.0)	34.3 (16.0;66.0)

Peak Post GnRH:	(n = 21)	(n = 16)	(n = 25)	(n = 13)	(n = 22)	(n = 23)	(n = 13)	(n = 13)
LH (mui/ml)	14.6 (4.8;35.1)	25.9 (7.5;83.3)	15.6 (4.8;67.0)	22.9 (7.3;83.3)	16.7 (4.8;67.0)	16.4 (4.8;83.3)	16.4 (5.5;40.3)	18.4 (5.5;40.3)
FSH (mui/ml)	3.0 (0.6;8.5)	5.4 (1.0;27.6)	3.2 (0.6;11.2)	5.6 (2.4;27.6)	3.2 (0.6;11.2)	3.2 (0.8;27.6)	4.9 (0.9;11.4)	4.9 (0.6;11.4)
TST (nmol/L)	3.35 (-5.25;21.6)	0.6 (-7.5;18.7)	3.3 (-7.5;21.6)	0.6 (-5.7;18.7)	2.9 (-7.5;21.6)	2.5 (-7.8;21.6)	1.6 (-0.6;16.8)	0.6 (-5.3;16.8)
								
E2 (pg/ml)	8.5 (-70.5;95.0)	13.0 (-45.5;49.0)	11.5 (-70.5;95)	3.5 (-45.5;49.0)	10.8(-70.5;95.0)	8.5 (-70.5;95.0)	8.5 (-45.5;49)	15.0 (-45.5;49)
IHB (pg/ml)	20.7 (-61.4;136.7)	7.7 (-54.8;188.7)	20.7(-61.4;188.7)	9.6 (-15; 67.4)	32.0 (-61.4;188.7)	20.7 (-61.4;188.7)	9.6 (-22.8;132.6)	9.6(-22.9;132.6)

Age (years)	48 (26;60)	44 (30;59)	49 (26;60)	41 (31;58)	46 (26;60)	48 (31;58)	49 (26;60)	45 (31;58)

Abstinence (days)	3.0 (0.2;8.5)	3.0 (1.4;14.3)	3.3 (5.0;10.4)	2.3 (0.2;14.3)	3.1 (2.2;8.5)	2.8 (1.3;14.3)	3.3 (0.2;14.3)	2.7 (1.3;8.2)

Table 1 compares the baseline and post peak GnRH challenge blood hormone levels between participants with normal and abnormal semen parameters. Sum post GnRH challenge hormone results were similar to the peak post GnRH challenge results presented, and are not shown. Age and abstinence was high. Median baseline E2 (normal range: 10–50 pg/ml) was consistently higher than the high end of the normal range in all paired groups, while baseline inhibin (normal range 100–400 pg/ml) were lower than the normal range for participants with abnormal semen counts and densities. Baseline LH (normal range: 1.5–9.2 miu/ml), FSH (normal range: 1.4–18.1 miu/ml) testosterone (8.4–28.8) and SHBG (normal range: 12.7–55 nmol/L) were all in the normal range for all groups.

### Multivariate relationships between semen parameters and endocrine measures

Table 2 summarises the significant multiple linear and logistic regression analysis relationships between semen parameters treated as outcomes and basal and post GnRH challenge blood endocrine levels adjusting for age, abstinence period, the presence of one or more physical abnormalities and fever in the last 2 months. Most of the significant relationships were with semen count and density.

**Table 2:** Significant multivariate associations between semen outcomes and endocrine measures

Semen Parameter	Endocrine Measure	Beta (SE)	P	Odds Ratio (CI)	R^2^
Abnormal semen count (< 20 × 10^6^)	Baseline inhibin		0.048	0.98 (0.96–0.99)	0.14
	Baseline FSH		0.021	1.69 (1.05–2.8)	0.22
	Post Peak GnRH LH		0.015	1.15 (1.03–1.3)	0.24
	Post Peak GnRH FSH		0.033	1.4 (1.0–1.9)	0.18
	Abnormally low baseline Inhibin (< 100 pg/ml)		0.032	8.2 (1.4–49.2)	0.18

Density	Abnormally low baseline inhibin	-69.5 (32.2)	0.039		0.05
	Baseline testosterone	-3.8 (1.83)	0.046		0.04
	Baseline inhibin	0.8 (0.3)	0.01		0.25

Abnormal Density (< 40 × 10^6^/ml)	Baseline FSH		0.028	1.85 (1.1–3.2)	0.14

Abnormal morphology (< 2%)	Abnormally high baseline E2 (> 50 pg/ml)		0.013	37.2 (2.1–655.2)	0.18

Abnormal motility (< 50%)	Abnormally high baseline E2		0.046	39.5 (1.1–1450)	0.34

Morphology	Abnormally high baseline E2	-1.27 (0.58)	0.036		0.21

* CI: 95% confidence interval

Covariates included in models: age, abstinence, presence of physical abnormality and fever in the last two months

There was a consistent positive relationship between low basal inhibin and low semen count and density (positive relationship between abnormally low semen count and abnormally low basal inhibin, negative relationship between abnormally low semen count and baseline inhibin, negative relationship between semen density and abnormally low baseline inhibin, positive relationship between semen density and baseline inhibin). For example, Table 3 shows that for the relationship between abnormally low semen count and abnormally low basal inhibin, the latter is the only significant covariate with a high prevalence odds ratio = 8.2.

**Table 3:** Logistic regression model investigating the relationship between abnormal semen count and abnormally low basal inhibin

Variable (unit)	Odds ratio (CI)*
**Abnormally low semen count (< 20 × 10^6^)**	
	
Abnormally low basal inhibin (< 100 pg/ml)	8.2 (1.4–49.2)
Age (years)	1.0 (0.92–1.1)
Abstinence (days)	1.0 (0.99–1.0)
Fever in the last 2 months	0.45 (0.07–3.1)
Physical abnormality	0.77 (0.1–6.3)
R^2 ^= 0.18, n = 34	

* CI : 95% Confidence interval

There were consistent positive, but weaker relationships (1> prevalence odds ratios < 2) between abnormally low semen count and density with baseline and post GnRH FSH levels (positive relationships between abnormally low semen count with baseline and post GnRH peak FSH, positive relationship between abnormally low density and baseline FSH).

Table 2 also shows that there were significant positive relationships between the prevalence of abnormally high basal estradiol with abnormally low morphology and motility. Table 4 shows a strong relationship between abnormally low morphology and abnormally high basal E2 (prevalence odds ratio = 37, CI:2–655).

**Table 4:** Logistic regression model investigating the relationship between abnormal morphology count and abnormally high basal estradiol

Variable (unit)	Odds ratio (CI)*
**Abnormally low semen morphology (score < 2)**	
	
Abnormally high basal estradiol (< 100 pg/ml)	37.2 (2.1–655.2)
Age (years)	1.1 (0.96–1.4)
Abstinence (days)	0.98 (0.96–1.0)
Fever in the last 2 months	14.8 (0.8–273.2)
Physical abnormality	0.4 (0.02–8.5)
R^2 ^= 0.4, n = 32	

• CI : 95% Confidence interval

### Multivariate relationships between endocrine hormones

Basal E2 and testosterone were not significantly related to basal LH. Basal LH and basal FSH were positively associated. (Adjusted = 0.42 (SD = 0.53), p < 0.0005, R^2 ^= 0.62, n = 49). Basal testosterone had a strong positive association (Adjusted = 2.58 (SD = 0.65), p < 0.0005, R^2 ^= 0.31, n = 49) with basal E2. Basal FSH had a significant negative relationship to basal inhibin (Adjusted  = 0.03 (SD = 0.007), p < 0.0005, R^2 ^= 0.27, n = 49)

The only significant relationships among basal and post GnRH challenge hormone levels of the same hormone when adjusted for age and baseline SHBG, were between basal FSH and both the peak post GnRH FSH ( = 0.269 (SD = 0.107), p = 0.017, R^2 ^= 0.11, n = 42) and the sum post GnRH FSH ( = 0.086 (SD = 0.038), p = 0.029, R^2 ^= 0.27), n = 26), and also between basal E2 and the sum post GnRH E2 ( = -0.167 (SD = 0.077), p = 0.042, R^2 ^= 0.11, n = 26).

Baseline SHBG was a significant positive predictor of baseline testosterone ( = 0.26 (SD = 0.46, p < 0.0005, R^2 ^= 0.39, n = 26), but a negative predictor of post GnRH challenge peaks of testosterone ( = -0.12 (SD = 0.42), p = 0.016, R^2 ^= 0.16, n = 26) and E2 ( = -0.51 (SD = 0.18), p = 0.006, R^2 ^= 0.14, n = 26) when adjusting for age.

Age was not a significant covariate of endocrine hormones, but was positively associated with baseline SHBG ( = 0.86 (SD = 0.36), p = 0.021, R^2 ^= 0.09, n = 49).

## Discussion

This study did not find consistent relationships between basal or post GnRH endocrine hormones and semen parameters amongst the study participants, but rather relationships between some semen parameters below WHO and Tygerberg criteria and some blood endocrines especially for values outside the reference range. Most of the expected physiological relationships between basal levels of hormones in the male hypothalamus-pituitary-gonadal axis, as well as relationships between basal hormones and SHBG were found in this study. With respect to relationships between blood basal and post GnRH levels of the same hormone, there were few significant relationships.

The positive relationship between semen count and basal inhibin levels and the negative relationship between semen count and basal FSH found in this study is consistent with the literature [3-5,7-10], suggesting that these hormones are markers of impaired spermatogenesis. This study has shown that abnormally low basal inhibin (< 100 pg/ml) strongly predicts abnormally low semen counts. No criteria could be set for FSH because blood basal levels in this study sample were well within the upper limit of normal (18.1 nmol/L). Jensen et al. [6] found criteria of basal inhibin < 80 pg/ml and FSH > 10 miu/ml to be 100% predictive of semen counts < 20 per millimeter. These criteria, however, seem more relevant for a clinical setting because in this study only one participant had such a low basal inhibin and high FSH, and he had a semen count of zero. The results in this study also suggest that abnormally high basal E2 (> 50 pg/ml) could be a marker of abnormally low semen morphology (< 2% normal) and motility (< 50%), possibly reflecting increased LH and testosterone release in the hypothalamus-pituitatry-testis-axis. The lack of a relationship of basal LH and testosterone with semen parameters and other hormones could be due to the wide range and fluctuations of these hormones [3].

The GnRH test was not found to add much information to that provided by basal blood endocrine levels with respect to relationships with semen parameters. Previously, Gerhard et al. [12] also did not find correlations between semen parameters and post GnRH hormone levels. The increased post GnRH blood LH and FSH (Table 2) levels found amongst those with abnormally low semen count (Table 2) in this study could reflect decreased negative feedback at the level of the hypothalamus-pituitary as a result of diminished testicular function.

Expected physiological relationships between basal LH and testosterone, and between basal LH and E2 were not found, but this may be due to the high variability of LH.

The few significant relationships between blood basal and post GnRH levels of the same hormone indicate that the basal level of a hormone do not reflect the response the hormone to GnRH stimulation. Besser [1] also did not find significant relationships between basal LH and FSH and peak post GnRH values in a study of English men. Peak LH and FSH levels after GnRH stimulation in this study were respectively 10 and 2 times more than basal levels, and consistent with those found in normal US and English males [1,2]. In the study of English men, testosterone and E2 were found to have a slow response to GnRH stimulation and did not change significantly over a 2 hour period [1], which explaines the weak response of testosterone, E2 and inhibin to GnRh stimulation found in this study.

Although most semen samples were collected via coitus interruptus (there were only four semen samples produced via masturbation), semen parameters measured in the study sample did not differ substantially from those measured in similar populations via masturbation [13].

An expected positive relationship between basal SHBG and age [27] was found. Age was not found to be a significant negative predictor of basal testosterone as expected [27,28], but this might have been due to the relatively high median age of the participants.

## Conclusion

The study has demonstrated the utility of serum basal inhibin, FSH and estradiol for predicting impaired male reproductive function. Abnormally low basal inhibin (< 100 pg/ml) was shown to be a marker of abnormally low semen count (< 40 million) and had a positive relationship with semen density. Abnormally high basal E2 (> 50 pg/ml) was shown to be a marker of abnormally low morphology (< 2%) and motility (< 50%). Baseline FSH had a negative relationship with semen count and density.

The GnRH challenge test appeared to add little value to information provided by baseline levels of reproductive hormones alone with regard to semen quality.

## Competing interests

The author(s) declare that they have no competing interests.

## Authors' contributions

MD have substantially participated in the conception, design, collection of data, analysis and interpretation of data in the study, and in drafting the manuscript and providing important intellectual content. JM have participated in the conception, design, analysis and interpretation of data in the study, and in drafting the manuscript and providing important intellectual content Both authors have read and appoved the final version of the paper submitted.
